# Research Progresses on the Physiological and Pharmacological Benefits of Microalgae-Derived Biomolecules

**DOI:** 10.3390/foods11182806

**Published:** 2022-09-11

**Authors:** Zhou Yu, Yan Hong, Kun Xie, Qingsheng Fan

**Affiliations:** 1 Functional Food Research Center, Sino German Joint Research Institute, Nanchang University, Nanchang 330006, China; 2 Pharmacological Research Laboratory, Jiangxi Institution for Drug Control, Nanchang 330006, China; 3 Medical College, Nanchang Institution of Technology, Nanchang 330006, China

**Keywords:** microalgae, anti-oxidation, anti-tumor, immunomodulatory, skin protection, metabolic diseases, neuroprotective effect, antiviral

## Abstract

Microalgae are a kind of photoautotrophic microorganism, which are small, fast in their growth rate, and widely distributed in seawater and freshwater. They have strong adaptability to diverse environmental conditions and contain various nutrients. Many scholars have suggested that microalgae can be considered as a new food source, which should be developed extensively. More importantly, in addition to containing nutrients, microalgae are able to produce a great number of active compounds such as long-chain unsaturated fatty acids, pigments, alkaloids, astaxanthin, fucoidan, etc. Many of these compounds have been proven to possess very important physiological functions such as anti-oxidation, anti-inflammation, anti-tumor functions, regulation of the metabolism, etc. This article aimed to review the physiological functions and benefits of the main microalgae-derived bioactive molecules with their physiological effects.

## 1. Introduction

Microalgae are a class of autotrophic single-celled organisms that are widely distributed on land and in oceans, and they are rich in nutrients and active in photosynthesis. Moreover, they are also one of the oldest organisms on earth [1]. At the same time, microalgae are also a class of organism that contain *chlorophyll A*. Their single frond cannot be divided into rhizomes and leaves [2]. According to relevant literature reports, about 50,000 microalgae have been recognized and about 30,000 have been investigated [3]. Microalgae are divided into cyanobacteria, green algae, golden algae, and red algae. Compared with other biomass resources, microalgae have the characteristics of not occupying arable land, a high biomass, a fast growth rate, strong adaptability, and they are easy to domesticate [4]. Another advantage of microalgae is that their cultivation does not require fertile soil. They have high photosynthetic efficiency, fast reproduction, strong environmental adaptability, and low water consumption. It is a new type of healthy and environmentally friendly food [5].

Microalgae contain a large number of nutrients. The protein content in microalgae can reach 2.4 times that which is in cheese, three times that which is in pork, four times that which is in flour and fish, and five times that which is in eggs [6]. Protein contents can be vastly different among microalgal species and strains, and it is substantially affected by the environments in which they are grown. Under cultivation, many species may contain high levels of protein, typically 40–60% of their dry matter [7], demonstrating that microalgae is a great source to meet human protein needs [8,9]. In addition to proteins, microalgae also contain a variety of active substances that possess many powerful pharmacological effects [10].

Some microalgae contain a large amount of polysaccharides, which usually have the functions of anti-tumor, anti-virus, anti-aging, enhancing immune activity, anti-radiation, anti-mutation, and they regulate blood lipid levels [11]. Other microalgae contain a lot of carotenoids which often have powerful antioxidant effects, for example: *Haematococcus pluvialis* is rich in astaxanthin [12]. Moreover, certain microalgae produce and accumulate significant amounts of omega-3 polyunsaturated fatty acids (PUFAs) such as eicosapentaenoic acid (EPA) and docosahexaenoic acid (DHA), which have been shown to improve the cognitive development in neonates. The Omega-3 PUFAs have been extracted and purified from microalgae *Seaweed* and *Microchlorococcus*. Here, we summarize some of the activities and benefits of microalgae-derived extracts and biomolecules.

## 2. Anti-Oxidation

Microalgae produce many molecules with antioxidant activities. The extract of *Mucidosphaerium* sp. Inhibits the inflammatory responses in the skin and joint cells, which attributed to its antioxidant properties. In dermal fibroblasts, dermal papilla cells, or synoviocytes, treatment with the extract can down-regulate the expression levels of interleukin-1β (IL-1β), IL-6, and tumor necrosis factor-α (TNF-α), which are cytokines that promote inflammation. Moreover, the extract was able to suppress the mRNA expressions of cyclooxygenase 2, matrix metalloproteinase 1 (MMP-1), MMP-3, and MMP-9, which are enzymes that are involved in extracellular matrix remodeling. This is associated with the reduction of total MMP protease activities. The intracellular reactive oxygen species (ROS) levels in synoviocytes that were treated with the extract from *Mucidosphaerium* sp. was reduced, and the antioxidant activity was estimated as 178.3 ± 0.9 μmol trolox equiv/g, which was higher than that in the control cells [13].

Many microalgae, such as *diatoms*, *dinoflagellates*, and *golden algae*, contain a high content of fucoxanthin, an orange-colored pigment, which has a strong antioxidant effect. It can eliminate free radicals and reduce the level of cellular oxidative stress, producing significant antioxidant effects [14]. As a result, the fucoxanthin treatment reduces the ROS content, and thus, this benefits cell survival and promotes further changes to the cellular processes such as ribosome biogenesis, lipid metabolism, and cell cycle regulation. It also affects signaling pathways that are mediated by Wnt and JAK-STAT pathways, which are involved in inflammation responses, and forkhead box class O proteins, which effectively prevent cell senescence [15,16].

*Chlamydomona*, a genus of Antarctic ice microalgae is a natural marine resource that is rich in polysaccharides. In mice that are treated with polysaccharides which are derived from *Chlamydomona,* the oxidative stress in the internal organs such as the liver and kidney and the serum markers of tissue damages such as amino transferases were significantly reduced. The serum levels of glutathione and glutathione peroxidase, and the contents of nitric oxide (NO) and malondialdehyde in the spleen and liver were also decreased after the polysaccharide treatment. All of these changes are associated with increases in the expression level of genes that are involved in the clearance of ROS, such as manganese superoxide dismutase, catalase, etc., and decreases in those involved in NO production, such as inducible NO synthase in the tissues and cells of those treated animals [17].

Wang et al. (in 2014) extracted 96.7% water-soluble polysaccharides from *Auricularia terrestris*. At 10 mg/mL, this polysaccharide extract demonstrated 92.7% of the scavenging activity against hydroxyl radicals, and a 0.445 reducing power for potassium ferricyanide [18]. Phycocyanin from *Nostoc Sphaeroides* has been shown to perform a scavenging activity that is used to eliminate hydroxyl and super anion free radicals and hydrogen peroxide in a dose dependent manner. In, in vivo experiments, the phycocyanin treatment can significantly reduce the production of malondialdehyde and the content of peroxides in the blood and liver and protect cell membranes and red blood cells [19]. *B. braunii*, a well-known microalga, can secrete a large amount of exopolysaccharide (EPS), which is a heteropolysaccharide with a high molecular mass, consisting of about 7–9% uronic acid, 2–4% protein, and 1.5–2% sulfate moieties. The monosaccharide composition of EPS is 52–54% galactosyl, 35–36% glucosyl, 9–10% arabinosyl (9.41–10.32%), and 1.8–2% fucosyl moieties. The crude EPS extract without deproteinization performed a stronger degree of antioxidant activity than the purified polysaccharide did. The 50% inhibitory concentration (IC50) of the crude EPS extract, which is the concentration at which half of the hydroxyl radicals are inhibited, is about 1.67 mg/mL, which is significantly lower than that of the deproteinized EPS, which has an IC50 of about 3.04 mg/mL [20].

Babić et al. (in 2016) found that the ethanol extract of *Calothrix* strongly inhibited the production of phenyl-1-picrylhydrazine free radicals. The IC50 of this ethanol extract is about 30 μg/mL. 

The extract from *Phormidium* can perform about 23 g/kg antioxidant activity, which is the highest in the plasma iron reduction ability test. It contains 12 kinds of polyphenols, mainly phenolic acids and flavonoid glycosides. The contents of the quinic acid, gallic acid, and vanillic acid are 502, 84.9, and 50 μg/g, respectively, which may be the main reason for its strong antioxidant activity. *Botryococcus braunii* releases significant quantities of EPS, which can be used in the preparations of regular and medicinal foods, and cosmetics. To obtain EPS, *B. braunii* SCS-1905 was extracted and precipitated in ethanol, and then, it was deproteinated. After that, column chromatography using a DEAE-cellulose matrix was used to purify the EPS [21].

*Haematococcus pluvialis* is a species of microalgae that is identified in fresh water. It is in the family Haematococcaceae, of the order Chlamydomonadales. This unicellular green alga can synthesize significant amount of carotenoids, which can achieve to 2–5% of the dry mass. The astaxanthin content in *haematococcus pluvialis* is very high. For this reason, *Haematococcus pluvialis* has the drawn attention of scientists who are interested in extracting and purifying naturally derived astaxanthin from this organism [22]. As a carotenoid, astaxanthin is not considered as a provitamin A (it could not be converted to retinal). However, it has various physiological activities in humans and animals. One activity in particular is its strong antioxidant effect, which can be used to treat fetal alcohol spectrum disorder, a problem that occurs when pregnant women are exposed to ethanol. The underlying mechanism is attributed to the astaxanthin-mediated reduction of oxidative stress and inflammation in hippocampus region, which lowers the expression of choline acetyltransferase in the nucleus of the medial septal cells, maintains the structural integrity of excitatory synaptic neurons, and coordinates the sensorimotor activities. Chiara Samorì et al., have shown that the astaxanthin treatment is able to promote follicle and oocyte maturation and development and can be used to attenuate Bisphenol A-induced toxicity in those cells [23]. The antioxidant mechanism of astaxanthin has been studied extensively by many groups. The data indicate that astaxanthin treatment can prevent the hydrogen peroxide-induced loss of the mitochondrial membrane potential and regulate the (phosphatidyl inositol 3-kinase)PI3K/Akt/Nrf2/HO-1 signaling pathway [24].

The antioxidant effect of microalgae is one of its aspects that was identified the earliest and studied the most extensively. The antioxidant mechanisms include the direct scavenging of free radicals, the chelation of pro-oxidative metal ions, the regulation of ROS-producing oxidase, and the enhancement of oxidative defense system. This well-known antioxidant function of the microalgae has the most mature application in the market. For example, astaxanthin is currently one of the most popular products that is derived from microalgae. Since oxidation occurs in every part of the body, it is related to the development of many diseases. However, information showing the antioxidant activities of microalgae products, improvement of disease progression, and the underlying molecular mechanisms is still limited. More in-depth research on the antioxidant effects of microalgae is anticipated in the future.

## 3. Anti-Tumor

The direct anti-tumor effects of *Spirulina* microalgae on glioblastoma have been studied in vivo. The brain samples of rats that were treated with *Sprirulina* microalgae were analyzed for the tumor size and gene expression levels. It appeared that the *Spirulina* treatment inhibited the tumor cell proliferation and induced cell death. In addition, the *spirulina* treatment significantly up-regulated the levels of miR-34a and miR25b micro-RNA that play a role in the PI3K/Akt/mTOR signaling transduction pathway [25]. Voráčová et al. (in 2017) studied the diazine compound nocuolin A (NoA) from *Nodularia* and *Anabaena*. NoA induced the apoptosis of cancer cells in a caspase-dependent manner. NoA at 0.7–4.5 μmol/L triggered cell death in several human cancer cells such as lung cancer A549 cells and H460 cells, pancreatic cancer BxPC cells, breast cancer MCF-7, JIMT1 and SKBR3 cells, glioma U87 and U251 cells, and ovarian cancer OVCAR5 cells. This antiproliferative activity of NoA was more potent in p53 mutant cell lines, suggesting the presence of a novel mechanism that is mediated by NoA, which could be a drug target for the treatment of tumors, independent of the p53 pathway [26].

Pham et al., found that extracts from three strains of *Candida oryzae* were cytotoxic to breast cancer MCF7 cells and colon cancer HCT116 cells, which had IC50 numbers at 47.8 to 232.0 mg/L [27]. Additionally, it has been shown that the extract from *Anabaena* sp. can induce apoptosis in cells of acute myeloid leukemia [28]. Studies showed that the daily oral administration of *Euglena gracilis* extract significantly attenuated the lung tumor growth, possibly by altering the gut microbiota. It significantly increased gut microbiota diversification, decreased the *Firmicutes* to *Bacteroidetes* proportion, and promoted the abundance of *Akkermansia* and *Muribaculum* [29].

Silva-Stenico et al., screened the anticancer activity of extracts that were obtained from 24 strains of microalgae, using CT-26 and 3LL cancer cells. The methanol extracts (MEs) of *C. raciborskii* CYP011K and *Nostoc* sp. perform an inhibitory activity in CT-26 and 3LL cancer cells [30]. *Chlorella* EPS was shown to inhibit the growth and the reduce viability of Hela cells, a classic cervical cancer cell line. The EPS treatment regulated the expression levels of differentially expressed genes and activated various cancer and apoptosis-related proteins, which include mitogen-activated protein kinase (MAPK), TNF-1α, PI3K, and Akt. The changes in the expression levels of 13 proteins that are involved in signal transduction pathways were found in cells that were treated with *Chlorella* EPS [31].

Lazado et al. (in 2019) investigated the anticancer activity of the extracts from microalga *Dunaliella* sp. These microalgae were allowed to grow in normal or stressed conditions, and then were extracted using different methods. The anti-tumor activities of the extracts were evaluated in 4T1 murine breast cancer cells. The experimental results demonstrated that the water extract performed a higher level of cytotoxic activity than the ethanol and hydroalcoholic extracts did. The extract from *Dunaliella* sp. which was cultured in a stress condition performed a higher level of anti-tumor activity than it did in a normal condition. This cytotoxic mechanism is apoptosis, not necrosis, due to the presence of DNA fragmentation and caspase-3 activation. The direct injection of the extract from the stressed *Dunaliella* sp. inhibited the tumor growth and enhanced the immune function in zebrafish. All these results were associated with the decreases in the expression levels of Arg-1, NOS-2, and COX-2 genes, which play roles in immunomodulation [32].

Due to their diversity of their distribution, habitat conditions, and species, the probability of identifying anti-tumor substances from microalgae is relatively high. In fact, many active compounds have been isolated from them. In the meantime, with the development of bioreactors, cell cultures, and bioengineering and other technologies, the research on the anti-tumor effects of microalgae has made meaningful progress, recently. However, it is still in the initial stage of screening for anti-tumor substances and their functional mechanisms. Additional well-designed clinical trials should be conducted to evaluate the efficacy and safety of bioactive compounds from microalgae such as polysaccharides in patients with cancers.

## 4. Immunomodulatory

Wu et al. (in 2021) isolated, fractionated, and purified the extracellular polysaccharide (CEP) of *Chlorella*, and analyzed the characterized activities of the fraction 4 (CEP4). CEP4 is made of sulfated heteropolysaccharides, which is composed of 41% glucosamine hydrocholoride, and 21% glucuronic acid moieties. In TAW264.7 macrophage cells, the CEP4 treatment significantly increased the production of cytokines such as TNF-α and IL-6, and NO. The effects were found to be associated with the increase in the cytokine gene expressions. In addition, the CEP4 treatment reduced the expression levels of receptor-interacting protein kinase 1 (RIPK1) and toll-like receptor 4 (TLR4), suggesting the reduction of TLR-signaling transduction. Other pathways mediating cytoplasmic DNA sensing, and lectin receptor (C-type) signal transductions were also affected. A further investigation of protein–protein interactions identified the key player proteins mediating the immunomodulation roles of CEP4 [33]. A sulfated polysaccharide with a molecular mass of around 197 kDa was also extracted from *Tribonema* sp. by Chen. The structure and function of this sulfated polysaccharide were studied. This heteropolysaccharide includes a significant proportion of the galactosyl group and exhibits immunomodulatory activities. Its treatment activates macrophages and stimulates the production of cytokines such as IL-6, IL-10, and TNF-α [34].

Patwal (in 2021) cultured *Scenedesmus acutus* in the presence of higher concentrations of K_2_HPO_4_ and MgSO_4_, and obtained an EPS with a higher glucose content than those that were cultured at lower K_2_HPO_4_ and MgSO_4_ concentrations. This EPS had immunostimulatory properties and stimulated the proliferation of human peripheral blood mononuclear cells (PBMCs) [35]. Qi et al., used hot water to extract polysaccharides from the green *Chlorella ellipsoidea* and subsequently separated the polysaccharides using anion exchange chromatography to obtain fractions for further analysis of their molecular characteristics and immunomodulatory activities. The results showed that some factions of the polysaccharides could stimulate productions of NO and cytokines in RAW264.7 macrophage cells. These fractions activated MAPK and nuclear factor-κB pathways, and in turn, stimulated the expressions of the cytokine mRNA [36].

*Dunaliella salina* secrete EPSs. The extraction, which was obtained using ethyl acetate, resulted in a fraction that modulated the proliferation rates of human PBMCs and RAW 264.7 macrophages in a healthy human being. The treatment with this ethyl acetate fraction enhanced the production of cytokines such as interferon-γ (IFN-γ) and TNF-α. The effects on cell proliferation are bimodal. At 250 and 500 mg/L, the fraction inhibits the cell proliferation, whereas at 1000 and 1500 g/L, it promotes the cell proliferation. When the PBMCs were treated with the fraction at 750 and 1000 mg/L, the TNF-α production was increased, suggesting that it has an immunostimulatory function. In RAW 264.7 macrophage cells, this faction inhibited cell proliferation with an IC50 of 691 mg/mL, and NO production with an IC50 of 630 mg/mL [37].

Alexandros (in 2021) investigated the effects of dietary supplementation with *Schizochytrium* in dairy goats, which had a regular diet of alfalfa hay and concentrate. The goats were separated into four groups, a control group with the concentrate without the addition of *Schizochytrium*, and three experimental groups that were fed 20, 40, or 60 g of *Schizochytrium* sp. in concentrate, per day. The blood samples were collected from the animals that were fed *Schizochytrium* for 20, 40, and 60 days and they subjected to the analysis of monocytes and neutrophils. Compared with those in the control group, the mRNA levels of the components in cytokine signal transduction pathways such as MYD88 and TLR4, pro-inflammatory cytokines such as IL-1β and TNF-α, and chemokines such as CCL5 and CXCL16 in monocytes of the supplementary groups were all reduced. Interestingly, when it was compared with those of the controls, the mRNA levels of MAPK and IL-1β in the neutrophils of the animals that were fed 40 and 60 g/d *Schizochytrium* were increased, whereas that in the group that was fed 20 g/d, it was decreased. These results indicate that *Schizochytrium* can modulate immunity in goats [38].

The effects of *Chlorella* powder on the cyclophosphamide-induced immunosuppression have been studied in mice. The results showed that *Chlorella* powder significantly increased the proliferation of lymphocytes, the phagocytosis of macrophages, and the cytotoxicity of natural killer cells. In addition, the treatment decreased the plasma levels of cytokines such as IL-12 and IFN-γ, which are associated with histological improvements in the spleen of cyclophosphamide-treated mice [39]. Others have studied the effects of *Chlorella* powder in humans, and they have reported that the oral administration of *Chlorella* powder for 4, 5, and 6 weeks significantly increased the secretory immunoglobulin A levels of the saliva [40]. Chae used ethanol to extract *Micractinium simplicissimum*, an Antarctic freshwater microalgae, and studied the effects of this extract on inflammation in RAW 264.7 macrophages cells. The treatment with the ethanol extract reduced the expression levels of IL-6, cyclooxygenase (COX)-2, inducible NO synthase, and TNF-α, suggesting that it can perform anti-inflammation activities. This was also associated with reduced NO production [41].

Bigagli used methanol to extract *Tisochrysis lutea* F & M-M36 and compared the effects of this ME with that of the equivalent concentration of fucoxanthin in RAW 264.7 macrophage cells that were treated with lipopolysaccharide (LPS). The treatment with this ME significantly inhibited the COX-2-dependent prostaglandin E2 (PGE2) production when it was compared with the control group. Both the ME and fucoxanthin showed the ability to stimulate the expression levels of IL-10 and heme oxygenase-1 (HO-1) and inhibit IL-6 and arginase 1 in those cells. Additionally, the ME treatment led to an increase in the expressions of NLR family pyrin domain-containing 3 (NLRP3) and mir-223, and a decrease in mir-146b when it was compared with the fucoxanthin treatment. These results indicate that the ME of *Tisochrysis lutea F & M-M36* has anti-inflammation functions, which can be attributed to the regulation of PGE2 production, and the expression levels of genes that are involved in immunity such as NLRP3 and mir-223. The active components in the ME, probably, are compounds with phenolic structures, which may work together with fucoxanthin to regulate inflammation [42].

Microalgae polysaccharides play important roles in immune regulation. Previously, studies of polysaccharides have been mainly focused on terrestrial organisms and macroalgae such as brown algae and red algae. Studies of polysaccharides from microalgae have been limited. Microalgal polysaccharides are abundant and structurally diverse, which leads to them performing distinct physiological activities. More studies of the relationships of the structures and these activities will be of great value.

## 5. Antibacterial Action

Molecules that perform antibiotic activities were isolated from microalgae for the first time by Pratt et al., in 1944. They isolated a fatty acid mixture from *chlorella*, which showed antibacterial and anti-autotoxic functions [43]. The antibacterial effects of fucoxanthin on 20 strains of bacteria were investigated by Karpinski et al., using an agar disc diffusion test after microdilution. Fucoxanthin was found to perform activities against *Staphylococcus epidermidis*, *Staphylococcus aureus,* and *Streptococcus agalactiae*, but it did not perform a bacteriostatic activity against strict anaerobic bacteria [44]. Sudomova et al., found that fucoxanthin exerted antibacterial effects on *mycobacterium tuberculosis* by inhibiting the UDP-galactopyranose mutase and arylamine-N-acetyltransferase activities, demonstrating the great potential of fucoxanthin for the treatment of tuberculosis [45]. Salvatore et al., studied two species of black sea algae, *Polysiphonia denudota* and *P. denudataf fragilis*, and found that they performed antibacterial activities, strongly [46].

Lauritano et al. (in 2018) investigated *Skeletonema tropicum* and *Chaetoceros pseudocurvisetus* for their ability to perform anti-tuberculosis activities. They reported that anti-tuberculosis activity only presented when these two microalgae were cultured under control and phosphate-limited situations, but not under a nitrogen starvation condition. The organic extracts of *Skeletonema tropicum* and *Chaetoceros pseudocurvisetus* performed an anti-tuberculosis activity, but not cytotoxicity against human cells [47]. In addition, the antibacterial substance EMTAHDCA was purified from *Nostoc* sp. MGL001 and was shown to be toxic to Dalton lymphoma cells with an IC50 of 372.4 ng/mL in a 24-h assay [48].

The antibacterial activity of the *dinoflagellate Amphidinium carterae* extract has also been evaluated, and the biologically active compounds were identified. It was found that the extract inhibited *Staphylococcus aureus* (Gram-positive), *Klebsiella pneumoniae*, and *Escherichia coli* (Gram-negative), *Mycobacterium tuberculosis,* and the fungus, *Aspergillus fumigatus* [49]. Danielli used solvents of different polarities to extract *Scenedesmus spinosa* and they found compounds that could perform an antibacterial activity. The extracts of water, dimethyl sulfoxide (DMSO), and acetone all inhibited the growth of *Bacillus subtilis*. Interestingly, the DMSO extract, but not the water or acetone extract, showed inhibitory effects on *Klebsiella pneumoniae* and *Escherichia coli* [50]. Levert A et al., identified three novel depsipeptides and thiosulfonamides from the French Polynesian marine *cyanobacteria Lyngbya majuscula*, which performed strong antimicrobial activity with the minimum inhibitory concentration of 6.7 μM [51].

Abdult et al. (in 2022) used methanol to extract *Chlorella sorokiniana*, *Chlorella* sp., and *Scenedesmus* sp., and tested this ME (methanol extraction) for the presence of activity against bacteria. The ME from *Chlorella* sp. demonstrated that it performed an antibacterial activity with effective concentrations at 0.312 to 6.25 g/L. The results of the MTT cell viability assay showed that these MEs were not toxic to Vero cells, demonstrating their safety. The major components of the ME from *Chlorella* were determined using a GC-MS. It contains 18.5% phenol, 18.3% hexadecanoic acid, 14.43% phytol, 13.69% 9,12-octadecadienoic acid, and 7.23% bicyclo heptane [52]. The antimicrobial activities of the crude diethyl ether extract of *Scenedesmus obliquus* and the fractions derived from it were evaluated by Diaa et al., The crude diethyl ether extract had an inhibition zone between 12.5 and 19.5 mm for all the bacteria that were tested and between 8.7 and 18.3 mm for the fungi that were tested. A further purification of the extract using column chromatography identified two fractions that performed high antimicrobial activities against the bacteria and fungi that were tested [53].

Daniela et al. (in 2022) have used acetone, chloroform, dichloromethane, ethyl acetate, methanol, and hexane to extract the biomolecules from *Chaetoceros muelleri* in an attempt to find antibacterial activities. The activities of these extracts against bacteria, including *Mycobacterium tuberculosis*, were tested. The hexane extract performed the strongest antibacterial activity against all the bacterial strains that were tested, showing a potent activity against *Mycobacterium tuberculosis*. The minimal inhibitory concentration was found to be at 100 μg/mL. On the other hand, the ME was not effective, with the IC50 being greater than 800 µg/mL, whereas the IC50 values of other extracts were between 267 and 142 μg/mL. Interestingly, the ME was the only one that did not show cytotoxicity in the Vero cells [54].

The main purpose of isolating compounds that can perform antibacterial biological activities from microalgae is to discover new drugs, new food preservatives, or new compounds for agricultural biocontrol applications. Most of the current studies of these antibacterial activities are conducted in vitro rather than *in vivo*. If more in vivo experiments were performed, the applications of the antibacterial effect of microalgae in health will be realized, increasingly.

## 6. Antiviral Activities

Deyab et al., have investigated the effects of extracts from five cyanobacteria, *Leptolinngbya boryana*, *Arthrospira platensis*, *Nostoc punctiforme*, *Oscillatoria* sp., and *Leptalynbya* sp. on two viruses, Coxsackie virus B3 (CVB3) and rotavirus (RV). The therapeutic indices (TI) of the MEs from *Leptolinngbya boryana*, *Arthrospira platensis*, *Nostoc punctiforme*, *Oscillatoria* sp., and *Leptalynbya* sp. were found to be 50.0, 30.0, 27.6, 16.6, and 20.0 for CVB3, respectively. In addition, the TI values of the extracts from *Arthrospira platensis* and *Oscillatoria* sp. were at 45 and 42.5 for RV, respectively. On the other hand, the TI values of the extracts from *Leptolinngbya boryana*, *Leptolyngbya* sp., and *N. punctiforme* were from 2.8 to 7 for RV, which are relatively low. The results of this study indicate that the extracts from these five microalgae performed antiviral activities against CVB3 and RV, suggesting the presence of natural and bioactive compounds that are useful for further study [55].

*Porphyra* polysaccharide can inhibit the replication of RSV, herpes simplex virus 1 (HSV-1), and CVB3 viruses. The underlying mechanism is to prevent the virus from entering the host cells by interacting with virus particles or host cells [56]. The extract of *Chlorella* can inhibit the replication of HSV-1 and prevent the attachment of viral particles to cell membrane for their entry [57].

Nakashima et al. (in 2017) administered *Euglena gracilis Z* to mice orally and observed that it significantly reduced the influenza virus titers in the lung and increased the levels of cytokines such as IFN-γ, IL-1β, etc. These results suggest that the antiviral activity of *Euglena gracilis Z* is performed through immune regulation [58]. *Euglena gracilis Z* treatment was able to relieve the symptoms in mice that were infected with the influenza virus. It effectively attenuated the infections of all the influenza viral strains that were tested, including those strains that are resistant to Oseltamivir and Amantadine, which are two anti-influenza medications. A further analysis of the mechanisms showed that the *Euglena gracilis* extract did not suppress viral replication. Either the pretreatment or the extended treatment with the extract was sufficient to reduce the viral titers in the cells that were infected by the influenza virus, suggesting that the target of the *Euglena gracilis* extract is the host cellular functions, rather than the viral replication per se [59]. The extract of *Spirulina* has been shown to inhibit HSV-1 infection with the same potency as acyclovir. In the in vitro experiments, the *Spirulina* extract was able to block the interactions between the vital particles and the host cells, which lead to reduced virial adhesion and entry to the cells. In clinical studies using a herpes exacerbation model, creams containing calcium spirulan and microalgae extracts were more effective in terms of prevention than creams with Acyclovir were [60]. The cytotoxicity of the cold-water extract of *Spirulina platensis* is very low. Therefore, doses of *Spirulina platensis* extract of up to 5000 mg/kg are safe and tolerated in model animals. Anti-influenza efficacy studies have shown that the *Spirulina platensis extract* inhibits the plaque formation of several influenza viral strains, even including the Oseltamivir-resistant strains. The extract of *Spirulina platensis* functions in the early stages of viral infection and production in the host cells, which improves the survival rates of mice that are infected with influenza and inhibits hemagglutination due to the influenza infection [61].

Fabregas et al. (in 1999) investigated the antiviral activities of water extracts of 10 marine microalgae, including *Dunaliella (D. tertiolecta)*, in vitro. The results showed that the water extracts significantly inhibited the replication of a viral haemorrhagic septicaemia virus and had a trend (but not one which was statistically significant) to inhibit the replication of the African swine fever virus. The active ingredients in the water extract of *D. tertiolecta* may be sulfated polysaccharides as polymer sulfated dextran (500 kDa) also has antiviral activity. Therefore, the responsible bioactive molecules in the water extracts of other microalgae might be also sulfated polysaccharides with a molecular mass ranging from hundreds to thousands Da, as experiments have shown that sulfuric acid glucan with masses from 5 kDa to 100 kDa are inactive [62]. Gupta et al., extracted dolastatin, debromo-dolastatin, anhydrodebromo-dolastatin, 3-methoxy dolastatin, and 3-methoxy debrominated dolastatin from *Trichodesmium erythraeum*. Three of the five, debromo-dolastatin, anhydrodebromo-dolastatin, and 3-methoxy debrominated dolastatin at concentrations of 0.1–10.0 μmol/L, exhibited a dose-dependent inhibitory effect on the chikungunya virus. The IC50 and selectivity indexes of debromo-dolastatin, and 3-methoxy debrominated dolastatin were estimated to be 1.3 and 2.7 μmol/L, and 10.9 and 9.2, respectively [63].

The antiviral activities of molecules that were derived from microalgae are related to many factors such as their growth stage, the habitat areas and environment that they are found in, the extract fractions, and the methods of examination. It appears that we can find new antiviral active substances from microalgae. However, their contents are generally very low, which are not sufficient to replace chemical and synthetic antiviral agents. With the progress of microalgae bioengineering technology, the applications of biological cultures, and genetic engineering and other technologies provide a simple and feasible way to obtain large amounts of antiviral substances from microalgae for their clinical uses.

## 7. Skin Protection

C-phycocyanin (C-PC) that has been extracted from *Spirulina* can be used to reduce skin wrinkles and prevent skin aging that is caused by UVB radiation. It has been speculated that the C-PC treatment facilitates the expression levels of MMP-1 and MMP-9, which participate in the skin tissue remodeling. In HaCaT cells, the C-PC treatment stimulated the expression levels of loricrin, filaggrin, and involucrin, which are the proteins that are protective to UV light damage [64,65].

The supplementation of astaxanthin has been shown to maintain the structural integrity of the skin, minimize wrinkle formation, and improve water retention in the skin. The use of astaxanthin also blocks the UV-induced damages in the skin, without causing any obvious detrimental effects. In clinical practice, a daily supplementation of 3 to 6 mg astaxanthin is protective to the aging process of the skin that is due to exposure to sun light [66]. When astaxanthin was added to PHK16-0b or HaCaT cells, the mRNA expression level of *AQP3* was significantly increased in a concentration-dependent way in both cell lines. AQP3 protein expression levels were also increased when astaxanthin was added to HaCaT cells. In the EpiSkin 3-D human epidermis cell culture model, the presence of astaxanthin is also increased the AQP3 expression. Moreover, the addition of astaxanthin facilities the permeability of glycerol in the EpiSkin 3-D model. The permeability of glycerol in the astaxanthin and glycerol group is higher than that in the glycerol-only group. Both the *AQP3* mRNA and its protein activity in the skin were elevated after the astaxanthin treatment [67]. Either oral or topical application of astaxanthin is sufficient to significantly restore skin moisture, improve elasticity, reduce wrinkle formation, and slow down the rate of skin aging [68].

Sun et al. (in 2021) reported that *Nannochloropsis* ethanolic extracts have a variety of skin protection functions. The ethanol extracts contain significant amounts of PUFAs such as EPA, carotenoids such as β-carotenes, astaxanthin, and zeaxanthin, and phenolic compounds. Based on the results of tyrosinase inhibitory activity, the ethanol extracts demonstrated multiple skin protective functions that include anti-melanin, moisturization, antioxidant, anti-inflammation, anti-photoaging, and anti-wrinkle. All these activities can help to restore cell viability after UV exposure [69].

With the help of a computer, Kose A et al. (in 2022) identified several peptides from the digested products of *S. platensis.* These peptides included P2 (MAACLR), P3 (RCLNGRL), P4 (RYVTYAVF), P5 (SPSWY), and P7 (AADQRGKDKCARDIGY). The authors tested the inhibitory activities of these peptides against tyrosinase of mushroom and B16-F10 mouse cells, and they reported that P5 and P7 were effective in reducing the tyrosinase activities. P5 was the most potent and had an IC50 of 12.1 µM for the mushroom tyrosinase. In addition to inhibiting tyrosinase, P2 and P3 can scavenge free radicals with an IC50 that is lower than 200 µM. For the tyrosinase of B16-F10 mouse cells, P5 and P7 performed inhibitory activities and had IC50 values at 48.9 and 34.2 µM, respectively. Interestingly, P4 can inhibit mushroom tyrosinase, but promoted melanin synthesis by three times in the B16-F10 cells. The results show that microalgal proteins can be considered as a source for biomolecules that can regulate melanin production [70].

The effects of the *Chlamydomonas reinhardtii* extract on melanin synthesis have been studied in B16F10, a human epidermal cell line, and other models mimicking human skin. The treatment with the *Chlamydomonas reinhardtii* extract dose-dependently inhibited α-melanocyte-stimulating hormone-induced melanin synthesis. In addition, this extract treatment also suppressed the mRNA and protein levels of tyrosinase, tyrosinase-related protein 1 and 2, and microphthalmia-related transcription factors. These inhibitory effects are associated with the reductions of the activation states of protein kinase A and the other up-stream kinases of melanin production [71].

Researchers have evaluated the effects of extracts from the freshwater microalgae *Scenedesmus rubescens* on the biomarkers of UV-irradiation-induced photodamage, such as decreased viability, decreased collagen content, hyperpigmentation, and sunburned cells, in, in vitro and ex vivo skin models. The extracts were shown to reduce the signs of cellular aging that were caused by UV exposure. The extract treatments increased the viability of dermal fibroblasts, rescued the content of dermal collagen, reduced the formation of sunburned cells, and inhibited the tyrosinase activity [72]. The protective effects of phycocyanin on UV-induced apoptosis have been studied in human dermal fibroblast cells and epidermal keratin-producing keratinocytes. A phycocyanin treatment induces the mRNA levels of the *HO-1* gene. In addition, it has been found that the phycocyanin treatment induced the nuclear translocation of nuclear factor (erythroid-derived 2)-like 2 (Nrf-2). Furthermore, the phycocyanin treatment attenuated UV-stimulated apoptosis and the expressions of p53 and Bax, and the activation of caspase-3. This treatment led to the phosphorylation of protein kinase C (PKC) α/β II. When PKC α/β II is specifically inhibited by its inhibitor Go6976, the phycocyanin-mediated induction of HO-1 expression is blunted. Furthermore, the small interfering RNA-mediated knockdown of HO-1 stimulated the cleavage of poly (ADP-ribose) polymerase 1, and the activation of caspase-3 after the pretreatment of phycocyanin. All of these results demonstrate that the phycocyanin-stimulated HO-1 expression needs an intact PKC α/β II-Nrf-2/HO-1 pathway, and the phycocyanin treatment attenuates UV-induced apoptosis in skin cells [73]. 

The uses of microalgae extracts have been shown to improve dermal health, which is mediated by the direct inhibition of several key players in the oxidative stress cascade, and inflammation, concurrently. This has been accepted as the main mechanism by which the microalgae extracts protect the skin from damages that are due to the exposure to sun light.

## 8. Treating Metabolic Diseases

The effects of polysaccharides from *Chlorella* and *Spirulina* on obesity have been studied in C57BL/6 mice that were fed a high-fat diet (HFD). The polysaccharides from these two microalgae were equally effective in attenuating body weight gain, disturbance of glucose and lipid metabolisms, inflammation, and a fatty liver in the HFD-fed mice. In addition, the microbiota in the gastrointestinal (GI) tract was also improved with the increase in the relative abundance of beneficial bacteria such as *Clostridium*, *Bacteroides*, and mollusks, and a decrease in the harmful ones such as *Actinomyces* and *Verrucobacterium* [74]. du Preez R et al., treated rats that were fed a high-carbohydrate and high-fat diet with *Nannochloropsis oceanica.* The treatment with *Nannochloropsis oceanica* promoted the retention of lean body mass in these animals, which is attributed to the elevated intake of dietary protein and an increase in the beneficial bacteria such as *Oxyphotobacter* in the GI tract [75].

The use of *Spirulina flatus* has been shown to prevent the rapid rise of blood glucose and insulin levels and correct the hepatic enzymes in streptozotocin-induced diabetic mice that are fed an HFD. The *Spirulina flatus* treatment also improved the plasma lipid profile and inflammatory markers such as adiponectin and TNF-α. The hepatic expression levels of sterol regulatory element-binding protein 1c (SREBP-1c), a master regulator of lipogenesis, is also reduced after the *Spirulina flatus* treatment, indicating its effect on the fatty liver. In addition, the *Spirulina flatus* treatment increased the hepatic expression levels of peroxisome proliferator-activated receptor α (PPARα), PPARγ coactivator-1α (PGC-1α), and mitochondrial transcription factor A, which are proteins promoting the mitochondrial biogenesis, indicating the elevation of energy expenditure in the treated animals [76].

Mayer et al. (in 2021) investigated the effects of *Tisochrysis lutea* on obesity and metabolic disturbances in rats. The supplementation of *Tisochrysis lutea* did not affect plasma alanine aminotransferase, indicating that were was tolerance to this treatment. On the other hand, the treatment reduced body weight, blood levels of glucose and insulin, and increased the levels of total and high-density lipoprotein (HDL-C), but not low-density lipoprotein cholesterol (LDL-C) and triacylglycerol (TAG) levels. The *Tisochrysis lutea* treatment also reduced the adipose tissue mass, blood leptin and TNF-α levels, and TAG and cholesterol contents in the liver, without any change in the blood IL-6, IL-4, and LPS concentrations. The expression level of IL-10 in the abdominal fat tissue was increased [77]. Preez et al., reported similar results of *Sargassum siliquosum* treatment and they found that it reduced abdominal fat deposition and liver fat droplet sizes [78].

Many studies have demonstrated that the dietary supplementation with *Arthrospira* significantly reduced fasting blood glucose (FBG), total cholesterol, and TAG levels, and it increased the HDL-C level without any change to the glycated hemoglobin A1c (HbA1C) and LDL-C. On the other hand, other studies have shown that the *Arthrospira* supplementation decreases FBG and HbA1C levels in animals with diabetes. In humans, the use of *Arthrospira* at a does that is less than 2 g per day for less than 2 months significantly reduced FBG. It appears that the *Arthrospira* supplementation is effective to reduce FBG, TAG, total cholesterol, and HDL-C levels in diabetic animal and humans, indicating its antidiabetic hyperlipidemic activities. The discrepancy of the effects of *Arthrospira* on HbA1C in the humans and animals is an interesting phenomenon, which requires more studies to reveal the underlying mechanisms that are involved in it [79].

Nacer et al. (in 2020) have studied the effects of *Nannochloropsis gaditana* supplementation on glucose metabolism in streptozotocin-induced diabetic rats. The animals were fed a diet with or without supplementation of 10% *Nannochloropsis gaditana* for 2 months. The results showed that the *Nannochloropsis gaditana* supplementation reduced their blood glucose and HbA1c levels and attenuated the amount of tissue oxidative stress and inflammation, which resulted in the improvements of functions in the liver and kidney of the diabetic rats [80].

Zhang et al. (in 2019) reported a peptide, LLVVYPWTQR, from *Chlorella pyenoidose* and studied its effects on pancreatic lipase. The synthetic peptide at 200 μg/mL is effective to inhibit the pancreatic lipase activity, which is attributed to its binding to the active site of the enzyme and the formation of hydrogen bonds by docking modeling. Furthermore, in 3T3-L1 cells, the synthetic peptide at 600 μg/mL significantly decreased the intracellular TAG accumulation (27.9%) to the same extent as 10 μM simvastatin (24.1%), the positive control drug. Further analysis of the mechanisms indicate that the peptide inhibits TAG accumulation and lipogenesis in 3T3-L1 adipocytes. The effect may be achieved by the mediation of major players in lipid metabolism such as SREBP-1c and cell energy sensing signaling proteins such as AMP activated protein kinase, suggesting its potential as a medication for obesity treatment [81].

Chlorella and diatoms have been shown to perform strong anti-advanced glycation end products activity, which may be attributed to astaxanthin and EPA [82]. Scholars have used ARPE-19 cells and rat models to study the underlying mechanism that is involved in them. The treatments with the extract of *Chlorella zofingiensis* and astaxanthin inhibited the formation of N-(1-Carboxymethyl)-L-lysine, which is mediated by the inhibition of intracellular oxidative stress [83]. In a rat model, the astaxanthin treatment increased the insulin sensitivity and ameliorated the diabetes’ development. The astaxanthin treatment (20 mg/kg body weight per day) was able to restore the altered activities of the submandibular salivary superoxide dismutase, catalase, and glutathione peroxidase to the normal levels in the diabetic animals [84].

Many of the metabolic diseases require long-term medications that often have certain side effects. Microalgae have therapeutic and preventive potentials and can be developed to become a new type of healthy foods for patients with metabolic diseases.

## 9. Neuroprotective Effect

The effects of the microalga *Aurantiochytrium* sp. on neural development and functions have been studied. The extract of *Aurantiochytrium* sp. was able to attenuate the β-amyloid-induced cytotoxicity and increase ATP production via the correction of metabolism in cells. The treatment promoted spheroid formation and increased the proliferation of β-III-tubulin positive neurons and glial fibrillary acidic protein (GFAP)-positive astrocytes. When the *Aurantiochytrium* sp. extract was orally administrated in the senescence accelerated mouse-prone 8 mice, a mouse model used for studying aging, the treatment reduced the escape latency in the Morris water maze experiment, improved their memory ability, and increased the BrdU incorporation in cells of the hippocampus dentate gyrus region. The effect may be associated with the rapid rises of BrdU + GFAP+ and BrdU + NeuN+, markers of stem cells and mature neurons, respectively [85].

*Spirulina* contains a plethora of nutrients and other bioactive molecules that are beneficial to brain health. Studies have shown that the *Spirulina* treatment relieved mental exhaustion and maintained the integrity of cerebrovascular vessel walls in response to endothelial damage and pressure. This helps to prevent and/or alleviate cerebrovascular disease. In addition, the *spirulina* supplementation appears to improve motor development, communication skills, and cognitive abilities in children experiencing malnutrition [86].

The fucoxanthin in microalgae also has certain neuroprotective effects. The fucoxanthin treatment not only inhibits the aggregation of β amyloid 1–42 (β amyloid1–42, Aβ1–42), but also regulates the activations of PI3K, Akt, and ERK-signaling transduction cascades, which prevents the Aβ oligomers-mediated loss of neurons and oxidative stress. The fucoxanthin treatment has been shown to attenuate Aβ1–42-induced neurotoxicity in SH-SY5Y cells [87]. It has been shown that the fucoxanthin treatment attenuated the secondary brain damages after a traumatic brain injury (TBI), which includes neurological deficit, brain edema, encephalopathy, and neuronal apoptosis in neurons. These beneficial effects are attributed to the activations of an antioxidant responsive element and autophagy pathways that are mediated by the Nrf-2 transcription factor. In animals, the fucoxanthin treatment enhanced the nuclear translocation of Nrf-2, which increases the expression levels of the HO-1 gene. This significantly improves the neurological functions, reduces infarction sizes, and decreases levels of proteins that are related to apoptosis in the brain [88,89]. Paudel et al., reported that fucoxanthin acted as an agonist for the activation of dopamine D3 and D4 receptors. This implies that fucoxanthin may be useful for the intervention of neurodegenerative disorders such as Parkinson’s disease. Taken together, fucoxanthin is worth further exploration as a biomolecule for the intervention of cranial nerve injury [90].

Özugur et al. (in 2022) reported that phototrophic microalgae can initiate oxygen production in the brain, which alleviated the oxygen deficit circumstance and rescued neuron viability in the pathological conditions that are associated with hypoxia [91].

DHA as a PUFA has been shown to activate Nrf-2 pathways and protect neurons in rats that are experiencing TBI. The DHA treatment retained neuronal functions, attenuated edema in the brain, and improved the cognitive abilities in the TBI-induced rats. These are associated with the reduction of nitrogenic oxygen species and ROS in the CA1 area and neurons of the hippocampus in the brain. It appears that the DHA treatment promotes the nuclear translocation of Nrf-2, which regulates the protein levels of HO-1 and NAD (P)H: quinone acceptor oxidoreductases 1 in hippocampal regions [92]. Rats with mild TBI have been treated with a dietary supplementation of omega-3 fatty acids and vitamin D for 30 days. Behavioral tests were performed at multiple time points to determine any improvement to their neuronal functions. The supplementation of omega-3 fatty acids and vitamin D successfully reduced the blood levels of total tau, glial fibrillary acidic protein, and ubiquitin c-terminal hydrolase L1 in those TBI rats [93].

The exact mechanism of the neuroprotective activities that are performed by microalgae is not fully understood. However, recent findings suggest that the protective mechanisms may include anti-oxidation, the inhibition of cholinesterase, the protection against β-amyloid aggregation, and the repairment of neuronal damage. The neuroprotective activities that are derived from some microalgal strains are mediated by DHA, carotenoids, polyunsaturated fatty acids, phenolic compounds, and sterols, etc.

## 10. Conclusions and Perspectives

Microalgae are autotrophic due to them performing photosynthesis and are found in a variety of places [94]. Macroalgae can be used to produce a large quantity of biomolecules with various physiological functions, which has drawn the attention of researchers in food, nutrition, biotech, and pharmacology [95]. Proteins, carotenoids, polysaccharides, PUFAs, vitamins, and color pigments for food processing, etc., can be extracted from microalgae. These compounds perform antioxidant, antimicrobial, antiviral, anti-tumor, neural protection, and anti-inflammation activities in various cells, animals, and humans [96,97].

Therefore, to make full use of the high value-added natural products in microalgae, new functions and resources need to be continuously investigated. It is necessary to explore suitable culture and induction conditions for different microalgae and to produce a large amount of the specific biomolecules. This also includes designing corresponding photoreactors and large-scale culture processors, accordingly. It is anticipated that more and more research will be conducted to fully explore the microalgae as a rich source for bioactive molecules.

## Data Availability

Data is contained within the article.
